# Prevention of bronchial fistulas after pneumonectomies for selected cavitary drug resistant lung tuberculosis

**DOI:** 10.3389/fsurg.2023.1151137

**Published:** 2023-03-30

**Authors:** Alexander V. Bazhenov, Andrei O. Mariandyshev, Sven G. Hinderaker, Einar Heldal, Igor Ya. Motus, Irina A. Vasilyeva

**Affiliations:** ^1^ Department of Thoracic Surgery, Ural Research Institute for Phthisiopulmonology—a Branch “National Medical Research Center of Phthisiopulmonology and Infectious Diseases”, Ekaterinburg, Russia; ^2^ Northern State Medical University, Arkhangelsk, Russia; ^3^ Northern Arctic Federal University, Arkhangelsk, Russia; ^4^ University of Bergen, Bergen, Norway; ^5^ LHL International, Oslo, Norway; ^6^ National Medical Research Center of Phthisiopulmonology and Infectious Diseases, Moscow, Russia

**Keywords:** tuberculosis, MDR-, XDR TB, pneumonectomy, muscle flap

## Abstract

**Background:**

The World Health Organization guidelines for management drug resistant tuberculosis include surgery as an additional method in selected cases. Pneumonectomies have higher risk of morbidity such as bronchial fistulas which may be prevented by bronchial stump covering. We compare two methods of bronchial stump reinforcement.

**Methods and materials:**

A retrospective single center follow-up study was done in 52 patients who underwent pneumonectomy for drug resistant pulmonary tuberculosis. Between 2000 and 2017 we performed pneumonectomies with pericardial fat reinforcement of bronchial stump in group 1 (*n* = 42), and between 2017 and 2021 in group 2 with pedicled muscle flap reinforcement group 2 (*n* = 10).

**Results:**

Bronchial fistulas occurred in 17/42 (41%) of patients group 1 and there was no fistula in group 2, and this was statistically different (Fisher's test *p* = 0.02). Post-operative complications were seen in 24/42 (57%) of the patients in Group 1, and 4/10 (40%) patients in Group 2 (Fischer's test *p* = 0.53). In group 1 positive bacteriology decreased from 74% to 24% just after surgery, and in group 2 it decreased from 90% to 10%, but this was not statistically different (Fisher's test *p* = 0.63). In group 1 no-one died the first month, but 8/42 (19%) died within a year; in group 2 one died within a month, and only this death (10%) within a year. This difference in case fatality was not statistically significant.

**Conclusions:**

The use of pedicle muscle flap for bronchial stump coverage during the pneumonectomies for destructive drug resistant tuberculosis can prevent severe postoperative fistulas and improve postoperative life.

## Introduction

Mycobacterium tuberculosis can develop resistance to any drug that is active against it. Multidrug resistance (MDR) is when the bacteria are resistant to both isoniazid and rifampicin; when there is additional resistance to any fluoroquinolone it is called pre-extensively drug resistance (pre-XDR); if the bacteria develops further resistance to bedaquiline or linezolid it is called XDR (1). Former USSR countries, China, DR Congo and India have the largest estimated numbers in the world of untreated MDR/RR-TB patients, and Russia ranks 8th on that list (1). In the 1990s the rate of TB in Russia was quite high, around 100 per 100,000 population, but it declined steadily to around 40 per 100,000 in 2020 (2–4). A high proportion of these TB patients had MDR-TB and many were in high-risk groups with like prisoners (2, 4).

The World Health Organization (WHO) has produced consolidated guidelines for drug-resistant tuberculosis treatment (5). The duration of treatment for MDR-TB is longer than for drug susceptible TB. Success rate of MDR-TB treatment in the world including patients with quinolone- resistance is below 60%, which is much lower than the minimum target for drug-sensitive TB at 85% (1, 6).

Surgical treatment can improve the outcomes for some MDR and pre-XDR TB patients (5, 7). WHO made a conditional recommendation for partial lung resection (lobectomy or wedge resection) as an adjunct to chemotherapy for rifampicin resistant TB and XDR-TB patients. A systematic review did not show that patients with radical pneumonectomy had better outcome than those who did not have surgery (7).

Pneumonectomy is used in TB patients with total lung destruction, threatened bleeding, spontaneous pneumothorax, and empyema with or without fistulas that could be fatal (8), and have a high risk of postoperative morbidity. Pneumonectomy for infectious diseases may lead to broncho-pleural fistulas with concomitant empyema in up to 30% of cases (8, 9); the case fatality may reach up to 70% (10, 11). To prevent such a threatening complication various methods of covering the bronchial stump are used: parietal flap, diaphragm flap, or intercostal flap (12–14).

To avoid broncho-pleural fistulas after pneumonectomies for drug resistant cavitary TB we have in our center in Russia covered the bronchial stump in two ways. From 2000 to 2017 we covered the stump with pericardial flaps, and since 2017 we applied a muscle pedicle flap (15). In this paper we compare the two methods.

The specific objectives of this study among drug resistant TB patients who had a pneumonectomy by two types of bronchial stump reinforcement, was to compare: (1) the proportion of broncho-pulmonary fistulas and other complications; (2) conversion at 12 months, (3) treatment outcomes and case fatality at 30 and 365 days.

## Materials and methods

### Study design

The study was a follow-up study comparing the outcomes of two types of pneumonectomies for pulmonary tuberculosis.

### Setting

The study was performed at the Ural Research Institute for Phtisiopulmonology (URIP)—a branch of the Federal State Budgetary Institution “National Medical Research Center for Phthisopulmonology and Infectious Diseases” of the Ministry of Healthcare of the Russian Federation; there are 300 beds. URIP serves 6 oblasts, 2 autonomic districts, 1 kray and 2 republics. The URIP itself is situated in the central part of the Russian Federation in Ekaterinburg, the central city of Sverdlovsk oblast. The size of this territory is about 2,400,000 square km, with an approximate population of 47 million. Most of the TB patients in our study were from the Sverdlovsk region.

While the overall TB incidence rate (per 100,000 population) declined in the Russian federation from 100 in 2000 to 77 in 2010 and 29 in 2020; in the Ural region the rates were 85 in 2000 and 2010, and 34 in 2020. The incidence rate (per 100,000 population) of any drug resistant TB in Russia was 22 in 2010 and 19 in 2020, and in Ural region 26 and 21 respectively (2–4). The treatment success of drug resistant TB was in Russia 56% in 2010 and 55% in 2020; in Ural 53% and 55% respectively. Surgery for TB (lung resections and thoracoplasties) was done in Russia in 6.4% of all TB patients in 2010 and 7.1% in 2020, and in Ural 6.7 and 6.2%, respectively (2–4).

When admitted to the department a multidisciplinary council (with a thoracic surgeon, anesthesiologist, radiologist, internist, and phthisiologist) evaluates the previous treatment, the indications for surgical treatment and the possible types of surgery.

### Study participants

The indications for pneumonectomies were one or more of the following: (1) large cavitary lesions, assessed as irreversible with spreading to all lung, with or without persisting excretion of *mycobacterium tuberculosis* (*m.tb.*), with or without drug resistant strains of expectorated *m.tb*. (2) Previous surgery, with cavitary lesions in the lung, persisting (or not) excretion of *m.tb.*, drug resistant strains *m.tb*. (3) Lung bleeding from the cavities in all parts of the lung with or without the presence of *m.tb.* in sputum, drug resistant *m.tb.* strains.

All the patients were divided into two groups according to the method of bronchial stump reinforcement. Group 1 were patients who had a bronchial stump reinforced with pericardial fat or just local tissues around the main bronchus: 36 patients had pericardial fat reinforcement, 6 patients had bronchial stump reinforcement with local tissue, all done between 2000 and 2017. Group 2 were patients who had a bronchial stump of the main bronchus reinforced with a pedicle of muscle flaps. We used latissimus dorsi muscle in 9 patients and a pectoralis major muscle in 1 patient. This procedure was done from 2017 to 2021.

After surgery we examined surgical specimen for mycobacterium tuberculosis using the smear microscopy, molecular-genetic and culture tests, and then monthly thereafter we examined sputum by smear microscopy and culture tests. Drug resistance was determined by molecular-genetic TB-TEST (16) and culture by BACTEC method (17).

All patients with drug resistant TB were treated in accordance the national treatment tuberculosis guidelines, in line with WHO recommendations of 2011 and 2018 (18, 19).

### Technical procedure two types of pneumonectomies

The surgery was performed in the operating room in lateral decubitus position under a total intravenous anesthesia with double-lumen tracheal tube intubation. Preoperative antibiotic prophylaxis was given according to the Institution protocols (ceftriaxone 2 g intravenous, 30 min before surgery). Every patient gave an informed consent for surgery and blood transfusion.

**Figure 1:**
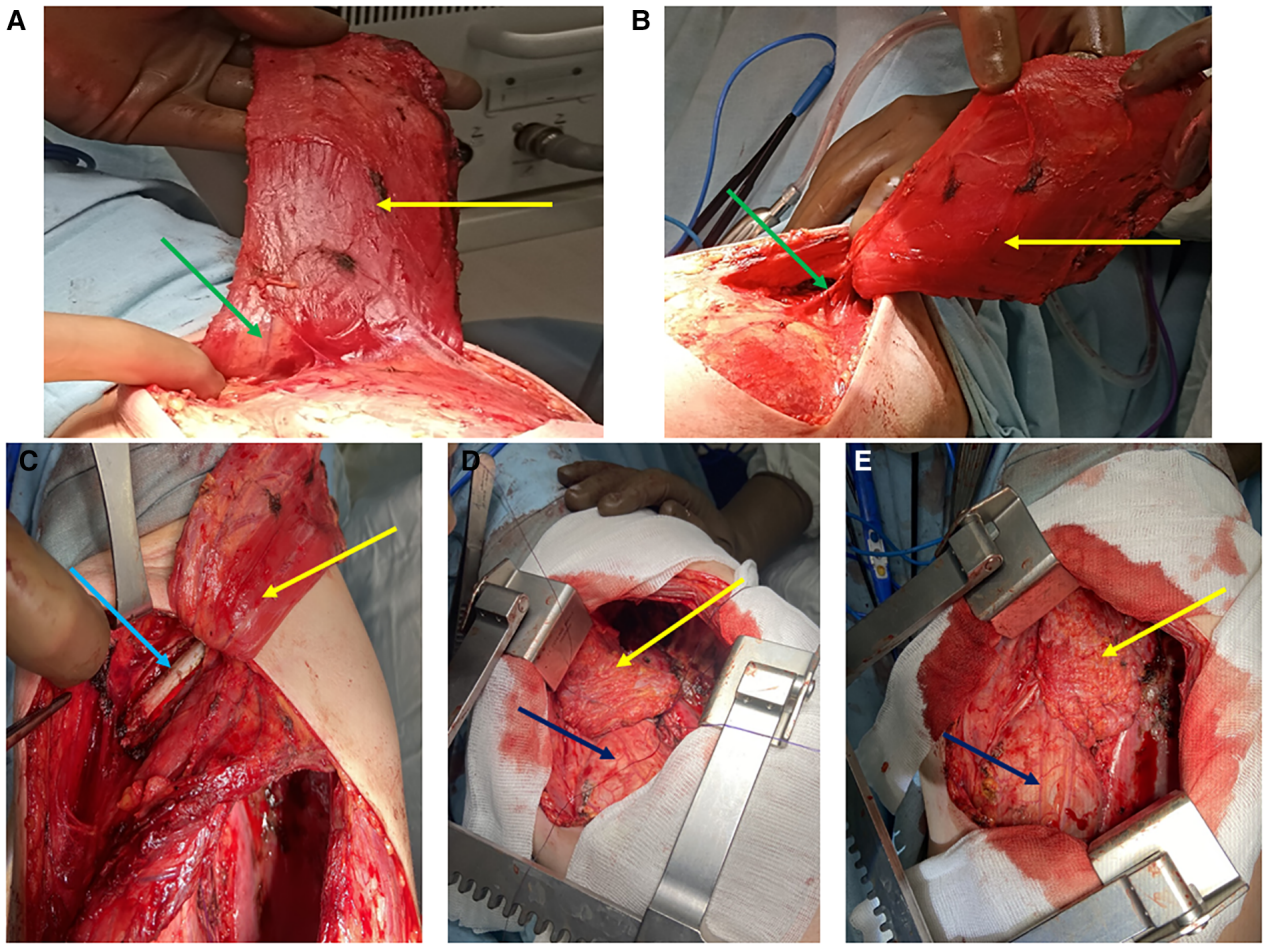
Stages of muscle flap transposition (**A, B** = latissimus dorsi muscle mobilization, **C**- resection of the rib to get access to the pleural cavity, **D** = fixation of the muscle flap to the bronchial stump and mediastinum, **E** = completion of the muscle flap fixation). Yellow arrow = muscle flap; green arrow = feeding vessels; blue arrow = rib need to be resected; dark blue = pericardium.

In order to mobilize the muscle flap we used the bipolar device LigaSure^tm^ of the ForceTriad^tm^ energy platform (ValleyLab, Covidien, Minneapolis, MN, USA). The muscle flap was placed into the pleural space *via* separate access in the area of 2nd or 3rd rib, made accessible by partial resection of one of those ribs in the length of 6–7 cm (15). The stages of the muscle flap intrathoracic transposition are shown in Figure 1.

In group 1 bronchial stump was closed by hand (16 patients) with interrupted absorbable sutures (with a long time degradation thread of 3/0) in the manner of “Cartilage to cartilage” (20) or by the stapler device (26 patients). In all the cases attempts were made to leave the stump of the bronchus no larger than 1 cm. In group 2 all the bronchial stumps (10 patients) were closed with cartilage to membrane using a 3-rows endo stapler (black cartridge) Endo GIA^tm^ (Medtronic, Minneapolis, MN, United States).

Pericardial fat was detached from the pericardium and affixed to the closed bronchial stump with 3–4 absorbable stitches of the monofilament biodegradable threads 4/0. The muscle flap was inserted into the pleural cavity and affixed to the previously closed bronchus with 4–5 interrupted stitches of 4/0 monofilament absorbable thread. We also fixed the muscle flap to the mediastinal tissues and in the area of intrathoracic entry of the flap with 3–4 interrupted stitches of absorbable thread 3/0. Close attention was paid to avoid twisting a vascular pedicle around its axis. The methodology of muscle harvesting and its introduction into the pleural cavity was described by Pairolero (15). The surgery was finished by drainage of the pleural cavity with a silicone tube. In group 1, we tried to remove drainage as soon as possible to ensure the absence of continuous intrapleural bleeding.

One pleural drain was used in group 1, while two pleural drains were used in group 2. Patients in group 2 also had one drainage placed into the bed of the harvested muscle. In group 2, pleural drainages were removed after pleural washing with antiseptic solutions for at least 2 weeks after surgery, and also after a minimum of two negative results of nonspecific bacterial growth in the pleural cavity. After removing the drainage from the bed of the muscle flap (when the amount of serous tissue fluid was 50 ml/day or less) patients wore the elastic chest bandage to compress the dissected skin flap to the chest wall to prevent development of seroma. TB drugs were restarted after four days beginning with parenteral drugs.

Before surgery the bronchial tree was examined by bronchoscopy for the presence of TB in the bronchi, and also after surgery once every ten days, during one month. It was performed later if clinical signs of broncho-pleural fistula were suspected.

### Definition of treatment outcome after surgery

Short-term (30 days) surgical outcome we defined as complications during first 30 days after surgery while still admitted to hospital, like broncho-pleural fistula, empyema, bleeding and other complications. Long-term surgical outcomes assessed at 12 months were culture conversion and death. Standard TB treatment outcomes after pneumonectomies were categorized according to WHO definitions and framework for tuberculosis (21): cured (bacteriology positive at start and negative at completion), completed (but no evidence of negative bacteriology), died (from any cause during TB treatment), failed (still positive bacteriology), lost-to-follow-up, transferred to another place.

### Data sources

The data was collected from the patients' clinical records, referral letters from the regional TB dispensaries, the Federal TB Register, and the “Tanatos” electronic system for death registration in Sverdlovsk oblast.

### Analysis and statistics

We analyzed by cross-tabulation and used non-parametric tests because of small sample: Fisher's exact test for categorical variables, and Mann-Whitney- test for continuous variables. The level of statistical significance was set at *p*-value ≤0.05.

## Results

The characteristics of the participants are shown in Tables 1A, 1B. In group 1 there were 42 patients, and in group 2 there were 10 patients. Patients with MDR were 57% in group 1% and 20% in groups 2, patients with pre-XDR were 33% in groups 1% and 50% in group 2, and patients with XDR none in group 1% and 20% in group 2. The groups were comparable for sex, age, height, presence of mycobacteria, patient address, x-ray changes, drug resistance, and previous invasive treatment. The surgery was planned in 36 of the 42 patients in group 1, and 6 were emergencies; all in group 2 were planned. In Group 1 there were 36 patients who had pericardial fat reinforcement and 6 patients who had bronchial reinforcement with local tissues; in Group 2 the reinforcement with pedicle muscle flaps was done in all 10 patients. The drug susceptibility test from the surgical material did not differ from the preoperative DST from sputum, except in group 2 where one patient had additional resistance to bedaquiline and linezolid in the surgical material. Emergency surgery was done in 6(14%) patients in group 1 and was caused by pulmonary bleeding proved by fibrobronchoscopy. Most of the surgical equipment was similar in the two periods, but in the later period there was more stable electricity.

**Table 1A:** Baseline characteristics of participants with drug resistant tuberculosis who had pneumonectomy, by type of surgical treatment, in URIP, Russia, 2000–2021.

		Group 1	Group 2	*p*-value*
Total		42 (100%)	10 (100%)	
Year of pneumonectomy		2000–2017	2017–2021	
Sex	Male	18 (43%)	7 (70%)	0.23
	Female	24 (57%)	3 (30%)	0.23
Age, median (range) years		35 (29;44)	40.5 (36;44)	0.35
Height, median (range) cm		170 (161;176)	176 (165;182)	0.35
Bodyweight, median (range) kg		59 (54;69)	61 (55;73)	0.5
BMI kg/m^2^		21.3	22.2	0.15
Smoking	Smoker	17 (41%)	3 (30%)	0.82
	non-Smoker	25 (59%)	7 (70%)	0.82
Former incarceration	Yes	7 (17%)	2 (20%)	
	No	34 (83%)	8 (80%)	
Side of pneumonectomy	Left lung	23 (55%)	8 (80%)	0.27
	Right lung	19(45%)	2(20%)	0.27

**Table 1B:** Pre-operative clinical characteristics of study participants with drug resistant tuberculosis who had pneumonectomy, in URIP, Russia, 2000–2021.

		Group 1	Group 2	*p*-value*
Total		42 (100%)	10 (100%)	
Former treatment, mean (range) months		59 (6–216)	108 (12–276)	0.002
Former invasive lung therapy		19 (45%)	4 (40%)	
Former lung surgery		11 (26%)	4 (40%)	0.62
Chest x-ray	Cavitary	34 (81%)	7 (70%)	0.71
	Pneumocirrhosis	8 (19%)	3 (30%)	0.71
Lung pathology	Cavitary	39 (93%)	8 (80%)	0.48
	Pneumocirrhosis	3 (7%)	2 (20%)	0.48
Sputum test for *m.tuberculosis*	Positive	31 (74%)	9 (90%)	0.52
	Negative	11 (26%)	1 (10%)	0.52
DST before surgery	Resistant to H	5 (12%)	1 (10%)	
	Resistant to R	4 (10%)	0 (0%)	
	Resistant to HR	21 (50%)	2 (20%)	0.17
	Resistant to HRFq	12 (29%)	6 (60%)	0.14
	Resistant to HRFqBqLzd	0 (0%)	1 (10%)	
DST in specimen	Resistant to H	2 (5%)	1 (10%)	
	Resistant to R	2 (5%)	0 (0%)	
	resistant to HR	24 (57%)	2 (20%)	0.08
	Resistant to HRFq	14 (33%)	5 (50%)	0.53
	Resistant to HRFqBqLzd	0 (0%)	2(20%)	

H, isoniazid; R, rifampicin; Fq, fluoroquinolones; Bq, bedaquiline; Lzd, linezolid.

* *p*-values from Fisher's exact test; small data prevent using test in some variables.

The outcomes are presented in Table 2. Post-operative complications were observed in 24 (57%) of the patients in group 1, and in 4 (40%) patients in group 2 (Fisher's test *p* = 0.53). In group 1 there were 17 out of 24 patients who had 2 or more complications per patient. Broncho-pleural fistulas occurred in 17(41%) in group 1 and none in group 2, and this difference was statistically significant (Fisher's test *p* = 0.02). From the 17 patients with broncho-pleural fistulas 10 (59%) patients were successfully treated by complex methods, including pleural space sanitation and fistula closure. There were 9 fistulas in 23 left sided pneumonectomies, and 8 fistulas in 19 right sided (Fisher's test *p* > 0.99); among those with fistula after left-sided pneumonectomy 3 out of 9 patients (33%) died; among right-sided fistula 3 out of 8 patients (38%) died (Fisher's test *p* > 0.99).

**Table 2:** Outcome of surgical treatment of participants with drug resistant tuberculosis who had pneumonectomy in URIP, Russia, 2000–2021.

			Group 1	Group 2	*p*-value*
Surgical procedure characteristics	Duration, (95% CI)		155 (120–174)	310 (285v363)	<0.001
	Blood loss, ml, (95% CI)		500 (341–688)	375 (313–500)	0.18
	Days in ICU, (95% CI)		1 (1–2)	4 (4–5)	<0.001
	Days drain, (95% CI)		3 (2–7)	30 (24–36)	<0.001
	Days before fistula		54 (12–210)	0	<0.001
	Bronchial stump closure	By hands	16 (38%)	0 (0%)	0.03
		By stapler	26 (62%)	10 (100%)	0.03
Complications	Intraoperative^a^		4 (10%)	1 (10%)	
	Postoperative empyema		18 (43%)	1 (10%)	0.1
	Postoperative fistula		17 (41%)	0 (0%)	0.02
	Postoperative bleeding		5 (12%)	1 (10%)	
	Postoperative other^b^		5 (12%)	2 (20%)	0.81
	Fistula site	Left bronchus	9 (39%)	0 (0%)	
		Right bronchus	8 (42%)	0 (0%)	
Treatment outcomes	Cured		26 (62%)	9 (90%)	0.18
	Completed		1 (2%)	0 (0%)	
	Success (cure + complete)		27 (64%)	9 (90%)	0.06
	Failed		2 (5%)	0 (0%)	
	Died		13 (31%)	1 (10%)	0.35
	Loss of follow up		0	0	
	Transfer		0	0	
Death	Died before 30 days		0 (0%)	1 (10%)	0.38
	Died before 1 year		8 (19%)	1 (10%)	0.88
	Died before 1 year, non-TB^c^		5(12%)	1(10%)	

^a^ Intraoperative complications: rupture of diaphragm during the mobilization of the lung; injury of the recurrent laryngeal nerve; rupture of the right pulmonary artery; injury of the left subclavian artery; spillage of the purulent sputum to the left main bronchus.

^b^ Postoperative complications “others”: wound infection; pneumonia; vein thrombosis; chylorrhea; arrythmia; esophageal fistula.

^c^ Non-tuberculosis cases of death within 1 year: pneumonia; bleeding from the eroded vessel in pleural cavity; suicide; heart failure; gastro-intestinal bleeding.

* *p*-values from Fisher's exact test; small data prevent using test in some variables.

The other complications were not different in the two groups. Surgical success with negative sputum result one month after pneumonectomies was observed in 27 patients (64%) in group 1 and 9 (90%) in group 2, but this was not statistically significant (Fisher's test *p* = 0.06) (Table 2). Right after surgery 12 patients (29%) were bacteriologically positive in group 1 and one patient (10%) in group 2.

The TB treatment outcomes in group 1 at final assessment of the 42 patients of whom 31 were initially sputum positive are also in Table 2, and 8 (19%) had died, 3 (7%) remained positive and 31 (74%) had negative bacteriology. In group 2 at final assessment of 10 patients of whom 9 were initially sputum positive 1(10%) had died and 9 (90%) had negative bacteriology. The sample was too small for good statistical comparison.

In group 1 no-one died the first month, but 8 (1-year case fatality rate 19%; 95% CI, 9%–33%) died within a year. In group 2 one patient died the first month, but no more patients died the first year, so 1-year case fatality rate was 10% (95% CI, 1%–40%). The causes of death in group 1 were as follows: 3 patients died from progression of TB in remaining lung; 1 patient bleeding from an eroded vessel; 2 patients bleeding from pulmonary artery; 1 patient suicide; 1 patient heart failure. In group 2 the only death was due to gastrointestinal bleeding.

## Discussion

Our study indicated that after pneumonectomies for drug resistant tuberculosis with reinforcement of the bronchial stump by pedicled muscle flap the proportion of patients who developed bronchial fistulas was significantly lower than among those reinforced with pericardial fat. The patients included in our study were clinically complicated with a long duration of their TB. Patients from both groups had previously been treated ineffectively; almost half of them with previous collapse therapy or lung resection. This made the pneumonectomies more complicated, especially during the lung mobilization by dividing the adhesions, and the dissection of the vessels and bronchi. Pneumonectomies in group 2 took more time because of delicate harvesting and insertion into the pleural cavity. De Palma (22) recommends inviting plastic surgeons to harvest the muscle flap, but we did not have that opportunity. However, the surgical team had specific practical training on cadavers to ensure skills in mobilizing and preparing the pedicle with muscle and vessels. We did not see muscle flap necrosis in our patients.

Several authors have reported successful draining of the contaminated or infected postpneumonectomy space (23). Most surgeons will let the tube remain in place to drain the contaminated or infected fluid until the cavity is clean and dry. At this point, some surgeons instill an antibiotic irrigation solution *via* this tube, and then the empty pleural cavity is filled with an antibiotic solution and the chest tubes are removed (23). In group 2 we used two large pleural drains (24) in order to wash the empty pleural cavity and to control the mediastinal shift. In group 2 silicone drains were inserted into the infected empty pleural space after surgery, and 5–7 days after surgery we started washing using the gravitational system. In our opinion the two large bore drains used by group 2 provided better conditions for cleaning the empty pleural cavity.

The data for the TB treatment outcomes after pneumonectomy showed treatment success of 64% in group 1% and 90% in group 2, but the difference was not statistically significant likely. The proportion who died (31% vs. 10%) was also not statistically significantly different in the two groups. Other studies on pneumonectomies in TB patients also have small samples and report treatment success of 60% (25), 70% (26), and 86% (27); the same studies reported case fatality rate 20%, 30% and 14%, respectively. Giller reported a case series of 766 pneumonectomies done between 1998 and 2016 where 40 (0.4%) died within 30 days after surgery, and with 93% sputum negative in MDR-TB and 92,1% sputum negative in XDR-TB patients (20). Yablonskii performed 39 pneumonectomies with no deaths within 30 days after surgery and all were sputum negative just after surgery (8, 28).

The proportion of patients with a positive smear microscopy in group 2 was 90% before and 11% right after surgery; whereas in group 1 surgery reduced bacillary load from 74% before and 29% right after surgery. The reduction in bacterial load in the patients seemed to be better in group 2, but in our small sample this was statistically not significant.

In our study we found that the duration of surgery in group 2 was twice as long as in group 2 because of preparation of the muscle flap; however, this did not result in higher blood loss. We can partially explain this observation by meticulous hemostasis performed during surgery. Also contributing may be the change in surgical technique. Previously a faster mobilization of the lung was attempted, followed by hemostasis. Now the lung is mobilized and hemostasis is done step by step, taking more time, but reduces blood loss. The surgical tools are not much changed from old to new method.

Some authors reported that the best approach to bronchial closure was to suture by hand (20, 29). Some old papers reported advantages of staplers (30, 31), another paper agreed but with specific reservations (32). In our material the bronchial stump closure in group 1 was done by suture in 16 and by stapler in 26; and in group 2 only by stapler. Broncho-pleural fistula developed in 5 (31%) of the 16 patients closed by hand and 12 (46%) of 26 patients closed by stapler; this was statistically not different (Fisher's test *p* = 0.5) but sample is small. A number of papers, old and new, came to similar conclusion about use of stapler (13, 29, 33, 34).

In group 1 the number of patients with fistulas was 17 (41%). All fistulas were confirmed by fibrobronchoscopy during the post-operative period. In group 2 there was no fistula. However, during performance of post-operative fibrobronchoscopy to control bronchial stump, we saw deficiency of staple suture with defects in 3/10 (30%) patients of group 2. The deficiency of the bronchial stump sutures did not depend on the size of bronchial stump coverage. It seems that infection (tuberculosis or other) could influence post-operative healing of the bronchial stump as reported previously by Björk, but it is still of current interest (35). Most of our 17 patients who developed fistula were initially treated with pleural space draining with a large silicone tube, and then several bronchoscopies were performed doing coagulation of the tissues around the fistula. Then if the pleural drainage was inefficient, we performed open window thoracostomy, allowing cleaning of the pleural cavity and plugging the fistula from outside. In some cases we used Amplatzer device to close the fistula, we also performed reamputation of bronchial stump, covering with the muscle flap (see Supplementary Table S1). Good results were reported using right thoracotomy access for the treatment of left main bronchus fistulas, but we did not use this method (22, 36). From these 17 patients with fistula 10 (59%) patients were successfully treated by complex methods of pleural space sanitation and fistula closure.

In a case series with 56 patients Wang stressed that for preventing broncho-pleural fistulas after pneumonectomy, it is reasonable to perform frozen section pathology on the resected bronchial stump to make sure that no mycobacteria remain in the bronchial stump (37). Our study may suggest that coverage of the bronchial stump with adequate amount of tissues, such as a muscle flap used for our participant in group 2, may be enough to prevent broncho-pleural fistula, without intraoperative frozen section examinations.

It is well known that reinforcing the bronchial stump is important after pneumonectomies performed for infectious diseases. However, what material is the best is still a question. Examples of materials used for stump reinforcement are pericardial flap (38), diaphragm and intercostal muscle flaps (39), omental flap (40), m.serratus anterior flap (41), vertical rectus abdominis myocutaneous flap (42), and latissimus dorsi muscle flap (13, 14, 43). Our results indicate that the use of latissimus dorsi pedicle muscle flap may be a good method for reinforcement of the bronchial stump, it is easy to perform and it prevents life threatening complications.

A strength of this study is its comprehensiveness, as we included all TB patients with pneumectomies of the URIP hospital during the period (2000–2021), which included all pneumonectomies in Sverdlovskaya oblast. But there were also patients from the other republics and oblasts from the catchment area of URIP, even though all territories conducted perform pneumonectomies. However, they would often send to the URIP hospital patients with a high risk of postoperative complications.

A limitation of our study was the before-after design where confounders not included in the study may influence results, including experience of the surgeons, post-surgical care, diagnostic and bacteriological tests, antimicrobial treatment given before and after surgery. MDR-TB treatment regimens in Russia were modified during the study period, as WHO published new recommendations on MDR treatment in 2011 (18), 2016, 2019 (19) and 2020 (5). Obviously, the use of new TB drugs and regimens could improve the outcomes of the later patients in Group 2. The total number of patients was very low in group 2, seriously reducing the statistical power of the study.

The lessons from our study could be used by centers of thoracic surgery among TB patients with drug resistance. The centers should be equipped with intensive care units and recovery units of highly experienced personal. This type of surgery needs an experienced, well-trained surgical team.

## Conclusion

Use of the pedicle muscle flap for bronchial stump coverage during pneumonectomies for destructive drug resistant tuberculosis can prevent severe postoperative fistulas and improve the lives of the patients.

## Data Availability

The data analyzed in this study is subject to the following licenses/restrictions: The datafile with no identifiers will be shared with publisher. Requests to access these datasets should be directed to Alexander Bazhenov ai0803@mail.ru.

## References

[1] World Health Organization. Global tuberculosis report 2021: Supplementary material. Geneva: World Health Organization (2022).

[2] Sterlikov SA Nechaeva OB Son OM Ponomarev SB. Industrial and economical indicators of TB work in 2019–2020. Analytic review of the basic indicators and statistic materials. Moscow: Russian research Institute of Health [in Russian] (2021).

[3] Son OM Nechaeva OB Sterlikov SA Kucheryavaya DA. Industrial indicators of TB service in 2009–2010. Moscow: Russian research Institute of Health (2011) [in Russian].

[4] Podgaeva VA. Epidemiological TB situation and activities of TB service at the Urals in 2017/ under a supervision of Skornyakov SN. Ekaterinburg: Ural Research Institute for Phthisiopulmonology (2018) [in Russian].

[5] World Health Organization. WHO Consolidated guidelines on tuberculosis. Module 4: treatment—drug-resistant tuberculosis treatment. Geneva: World Health Organization (2019). 32603040

[6] Sterlikov SA Vasilyeva IA Testov VV. Efficacy of tuberculosis patients treatment: problems and the ways of solving. Tuberculosis and Lung Diseases. (2015) 6:146–7 [in Russian].

[7] Harris RC Khan MS Martin LJ Allen V Moore DAJ Fielding K The effect of surgery on the outcome of treatment for multidrug-resistant tuberculosis: a systematic review and metaanalysis. BMC Infect Dis. (2016) 16:262. 10.1186/s12879-016-1585-0 27283524 PMC4901410

[8] Yablonskii PK Kudriashov GG Avetisyan AO. Treatment of pulmonary Tuberculosis. Thorac Surg Clin. (2019) 29:37–46. 10.1016/j.thorsurg.2018.09.003 30454920

[9] Klotz LV Eberhardt R Herth FJF Winter H. Interventional treatment of tracheopleural and bronchopleural fistulas. Chirurg. (2019) 90(9):697–703 [in German]. 10.1007/s00104-019-0977-2 31161248

[10] Bouchikh M Achir A Caidi M Aziz SE Benosman A. Role of pulmonary resections in management of multidrug-resistant tuberculosis. A monocentric series of 29 patients. Rev Pneumol Clin. (2013) 69(6):326–30 [in French]. 10.1016/j.pneumo.2013.09.002 24210152

[11] de Perrot M Spiliopoulos A. Postpneumonectomy bronchopleural fistula. Ann Thorac Surg. (1999) 68(5):1886–7. 10.1016/s0003-4975(99)01012-7 10585090

[12] Sherwood JT Mitchell JD Pomerantz M. Completion pneumonectomy for chronic mycobacterial disease. J Thorac Cardiovasc Surg. (2005) 129(6):1258–65. 10.1016/j.jtcvs.2004.12.053 15942565

[13] Shiraishi Y Nakajima Y Katsuragi N Kurai M Takahashi N. Pneumonectomy for nontuberculous mycobacterial infections. Ann Thorac Surg. (2004) 78(2):399–403. 10.1016/j.athoracsur.2004.02.103 15276484

[14] Pomerantz BJ Cleveland JC Jr Olson HK Pomerantz M. Pulmonary resection for multi-drug resistant tuberculosis. J Thorac Cardiovasc Surg. (2001) 121(3):448–53. 10.1067/mtc.2001.112339 11241079

[15] Pairolero PC Arnold PG Piehler JM. Intrathoracic transposition of extrathoracic skeletal muscle. J Thorac Cardiovasc Surg. (1983) 86(6):809–17. 10.1016/S0022-5223(19)39056-7 6645586

[16] Vakhrusheva DV Eremeeva NI Umpeleva TV Belousova KV. The experience of use of TB-TEST technology (Biochip – IMB, Russia) in diagnostic algorithm. Tuberc Lung Dis. (2017) 95(10):29–35 [in Russian]. 10.21292/2075-1230-2017-95-10-29-35.

[17] Chernousova LN Sevastyanova EV Larionova EE Smirnova TG Andreevskaya SN Popov SA Federal clinical recommendation for microbiology and molecular-genetic diagnostic of tuberculosis. Moscow: Russian Society of Phthisiatrists (2015) [in Russian].

[18] World Health Organization. Guidelines for the programmatic management of drug-resistant tuberculosis (2011) update. Geneva: World Health Organization (2011). 23844450

[19] World Health Organization. Consolidated guidelines on drug-resistant tuberculosis treatment. Geneva: World Health Organization (2019). 30946559

[20] Giller DB Giller BD Giller GV Shcherbakova GV Bizhanov AB Enilenis II Treatment of pulmonary tuberculosis: past and present. Eur J Cardiothorac Surg. (2018) 53(5):967–72. 10.1093/ejcts/ezx447 29244096

[21] World Health Organization. Definitions and reporting framework for tuberculosis-2013 revision. Geneva: World Health Organization (2014).

[22] De Palma A Maruccia M Di Gennaro F. Right thoracotomy approach for treatment of left bronchopleural fistula after pneumonectomy for tubercolosis. Gen Thorac Cardiovasc Surg. (2020) 68(12):1539–42. 10.1007/s11748-020-01307-4 32036566

[23] Wolfe WG Lewis CW. Control of the pleural space after pneumonectomy. Chest Surg Clin N Am. (2002) 12(3):565–70. 10.1016/s1052-3359(02)00021-2 12469487

[24] Cooke DT David EA. Large-bore and small-bore chest tubes: types, function, and placement. Thorac Surg Clin. (2013) 23(1):17–24. 10.1016/j.thorsurg.2012.10.006 23206714

[25] Dravniece G Kain KP Holtz TH Riekstina V Leimane V Zaleskis R. Adjunctive resectional lung surgery for extensively drug-resistant tuberculosis. Eur Respir J. (2009) 34:180–3. 10.1183/09031936.00047208 19567603

[26] Owen RM Force SD Pickens A Mansour KA Miller DL Fernandez FG. Pneumonectomy for benign disease: analysis of the early and late outcomes. Eur J Cardiothorac Surg. (2013) 43:312–7. 10.1093/ejcts/ezs284 22611143

[27] Riskiyev A Ciobanu A Hovhannesyan A Akopyan K Gadoev J Parpieva N. Characteristics and treatment outcomes of patients with Tuberculosis receiving adjunctive surgery in Uzbekistan. Int J Environ Res Public Health. (2021) 18(12):6541. 10.3390/ijerph18126541 34204519 PMC8296362

[28] Yablonskiy P Vasilev I Kirjuhina L. Immediate results of pneumonectomies in patients with unilateral localization of destructive pulmonary tuberculosis. Results of the prospective, non randomized study. Medicinskij Alliance. (2017) 4:103–11 [in Russian].

[29] Naidoo R. Active pulmonary tuberculosis: experience with resection in 106 cases. Asian Cardiovasc Thorac Ann. (2007) 15(2):134–8. 10.1177/021849230701500211 17387196

[30] Hakim M Milstein BB. Role of automatic staplers in the aetiology of bronchopleural fistula. Thorax. (1985) 40(1):27–31. 10.1136/thx.40.1.27 3969652 PMC459973

[31] Betts RH Takaro T. Use of a lung stapler in pulmonary resection. Ann Thorac Surg. (1965) 1:197–202. 10.1016/s0003-4975(10)66743-4 14298312

[32] Ravitch MM Steichen FM Fishbein RH Knowles PW Weil P. Clinical experiences with the Soviet mechanical bronchus stapler, (UKB-25). J Thorac Cardiovasc Surg. (1964) 47:446–54. 10.1016/S0022-5223(19)33578-0 14180749

[33] Vashakidze S Gogishvili S Nikolaishvili K Dzidzikashvili N Tukvadze N Blumberg HM Favorable outcomes for multidrug and extensively drug resistant tuberculosis patients undergoing surgery. Ann Thorac Surg. (2013) 95(6):1892–8. 10.1016/j.athoracsur.2013.03.067 23642435 PMC3728898

[34] Zakkar M Kanagasabay R Hunt I. No evidence that manual closure of the bronchial stump has a lower failure rate than mechanical stapler closure following anatomical lung resection. Interact Cardiovasc Thorac Surg. (2014) 18(4):488–93. 10.1093/icvts/ivt502 24351508 PMC3957281

[35] Bjork VO. Suture material and technique for bronchial closure and bronchial anastomosis. J Thorac Surg. (1956) 32:22–7. 10.1016/S0096-5588(20)30426-8 13332688

[36] Moreno P Lang G Taghavi S Aigner C Marta G De Palma A Right-sided approach for management of left-main-bronchial stump problems. Eur J Cardiothorac Surg. (2011) 40(4):926–30. 10.1016/j.ejcts.2010.10.044 21388823

[37] Wang H Lin H Jiang G. Pulmonary resection in the treatment of multidrug-resistant tuberculosis: a retrospective study of 56 cases. Ann Thorac Surg. (2008) 86:1640–5. 10.1016/j.athoracsur.2008.07.056 19049764

[38] Taghavi S Marta GM Lang G Seebacher G Winkler G Schmid K Bronchial stump coverage with a pedicled pericardial flap: an effective method for prevention of postpneumonectomy bronchopleural fistula. Ann Thorac Surg. (2005) 79:284–8. 10.1016/j.athoracsur.2004.06.108 15620959

[39] Asamura H Naruke T Tsuchiya R Goya T Kondo H Suemasu K. Bronchopleural fistulas associated with lung cancer operations. Univariate and multivariate analysis of risk factors, management, and outcome. J Thorac Cardiovasc Surg. (1992) 104(5):1456–64. 10.1016/S0022-5223(19)34643-4 1434730

[40] Nasirov FG Porkhanov VA Khudajbergenov SN Eshonckhodzhaev OD Irisov OT. Improved method of bronchial stump plasty of the main bronchus after pneumonectomy. Khirurgiia (Mosk). (2010) 5:53–5 [in Russian]. 20559213

[41] Kang MW Kim HK Choi YS Kim K Shim YM Koh WJ Surgical treatment for multidrug-resistant and extensive drug-resistant tuberculosis. Ann Thorac Surg. (2010) 89(5):1597–602. 10.1016/j.athoracsur.2010.02.020 20417785

[42] Kynta RL Sun N Saikia MK. Muscle flaps in pulmonary infections: a case series from Northeast India. Asian Cardiovasc Thorac Ann. (2020) 28(8):488–49. 10.1177/0218492320949074 32762245

[43] Kir A Inci I Torun T Atasalihi A Tahaoglu K. Adjuvant resectional surgery improves cure rates in multidrug-resistant tuberculosis. J Thorac Cardiovasc Surg. (2006) 131:693–6. 10.1016/j.jtcvs.2005.09.033 16515925

