# Supplementary material for: Prevention of bronchial fistulas after pneumonectomies for selected cavitary drug resistant lung tuberculosis
Source: Front Surg. 2023 Mar 30;10:1151137. doi: 10.3389/fsurg.2023.1151137 (PMC10097893; doi:10.3389/fsurg.2023.1151137)
Supplement: Supplementary file 1 [file Table1.docx]

**Supplementary Table 1. Management and outcomes of 17 broncho-pleural fistulas after pneumonectomies, Russia, 2000-2021.**

| **Sex** | **Age** | **Side** | **Method for managing fistula** | | | | | | | | | **Outcome** |
| --- | --- | --- | --- | --- | --- | --- | --- | --- | --- | --- | --- | --- |
|  |  |  | PD^1^ | OWT^2^ | TMP^3^ | EndoTreat^4^ | TTR^5^ | AD^6^ | RB^7^ | PMF^8^ |  | |
| Male | 42 | Left | + | + |  |  | + |  |  |  | Success | |
| Male | 52 | Right | + | + | + |  |  | + |  |  | Success | |
| Male | 28 | Left | + | + |  |  | + |  |  |  | Success | |
| Male | 37 | Right | + | + | + |  | + | + |  |  | Fail | |
| Male | 24 | Left | + |  |  | + |  |  |  |  | Success | |
| Male | 32 | Left | + |  |  | + |  |  |  |  | Success | |
| Female | 26 | Left | + |  |  | + |  |  |  |  | Fail | |
| Female | 22 | Left | + |  |  | + |  |  |  |  | Fail | |
| male | 38 | Right | + | + |  |  |  |  |  |  | Fail | |
| Male | 37 | Left | + |  |  | + |  |  |  |  | Success | |
| Female | 32 | Right | + | + | + |  | + |  |  |  | Success | |
| Female | 63 | Right | + | + |  |  |  |  |  |  | Fail | |
| Male | 42 | Right | + | + |  |  |  | + |  | + | Success | |
| Female | 43 | Left | + | + |  |  |  | + |  |  | Fail | |
| Male | 59 | Right | + |  |  | + |  |  |  |  | Success | |
| Female | 49 | Right | + |  | + | + |  |  |  |  | Success | |
| Male | 44 | Left | + | + |  |  |  |  | + |  | Fail | |

Footnotes: 1. PD pleural drainage; 2. OWT Open window thoracostomy; 3.TMP Thoracomyoplasty; 4. EndoTreat Endoscopic treatment; 5.TTR transsternal transpericardial resection of bronchus with fistula; 6. AD Amplatzer device; 7. RB Reamputation of the bronchus with broncho-pleural fistula via ipsilateral thoracotomy; 8. PMF Pedicle muscle flap.
